# Microbial Consortia for Effective Biocontrol of Root and Foliar Diseases in Tomato

**DOI:** 10.3389/fpls.2021.756368

**Published:** 2021-11-05

**Authors:** Zhivko Minchev, Olga Kostenko, Roxina Soler, María J. Pozo

**Affiliations:** ^1^Business Unit Microbiology, Agronomical Development Department, Koppert Biological Systems, Berkel en Rodenrijs, Netherlands; ^2^Department of Soil Microbiology and Symbiotic Systems, Estación Experimental del Zaidín, CSIC, Granada, Spain

**Keywords:** arbuscular mycorrhiza, biocontrol, disease suppression, microbial consortia, SynCom, *Trichoderma*, plant-growth promoting rhizobacteria, induced systemic resistance

## Abstract

The use of beneficial microorganisms for the biological control of plant diseases and pests has emerged as a viable alternative to chemical pesticides in agriculture. Traditionally, microbe-based biocontrol strategies for crop protection relied on the application of single microorganisms. However, the design of microbial consortia for improving the reliability of current biological control practices is now a major trend in biotechnology, and it is already being exploited commercially in the context of sustainable agriculture. In the present study, exploiting the microbial library of the biocontrol company Koppert Biological Systems, we designed microbial consortia composed of carefully selected, well-characterized beneficial bacteria and fungi displaying diverse biocontrol modes of action. We compared their ability to control shoot and root pathogens when applied separately or in combination as microbial consortia, and across different application strategies that imply direct microbial antagonism or induced systemic plant resistance. We hypothesized that consortia will be more versatile than the single strains, displaying an extended functionality, as they will be able to control a wider range of plant diseases through diverse mechanisms and application methods. Our results confirmed our hypothesis, revealing that while different individual microorganisms were the most effective in controlling the root pathogen *Fusarium oxysporum* or the foliar pathogen *Botrytis cinerea* in tomato, the consortia showed an extended functionality, effectively controlling both pathogens under any of the application schemes, always reaching the same protection levels as the best performing single strains. Our findings illustrate the potential of microbial consortia, composed of carefully selected and compatible beneficial microorganisms, including bacteria and fungi, for the development of stable and versatile biological control products for plant protection against a wider range of diseases.

## Introduction

A plethora of soil-borne microorganisms live associated with plant roots, and although some are detrimental, others provide important benefits to the host plant, from improved nutrition through growth and protection against multiple abiotic and biotic stresses (Bakker et al., 2018). Nowadays soil microbes are considered key players in modern crop management programs aiming to increase sustainability in agriculture (Barea, 2015; Trivedi et al., 2017; Compant et al., 2019). The use of plant beneficial microorganisms as biological control agents (BCAs) of pests and diseases emerges as a viable alternative to the abusive use of agrochemicals (Ab Rahman et al., 2018; Rändler-Kleine et al., 2020). A strong increase in registered microbial biocontrol agents worldwide in recent years serves as good evidence (van Lenteren et al., 2017). Yet, while the use of insects and mites to control pests is well established and used in practice for decades, microbes to control pests and diseases are in an earlier developmental phase (Mitter et al., 2019).

The ability of microorganisms to control pests and diseases has been well documented, but the variability of results often recorded under field conditions is one of the major challenges for wider adoption in agriculture (Trivedi et al., 2017; Mitter et al., 2019). Originally, biocontrol research focused on the application of single microorganisms (Sarma et al., 2015; Trivedi et al., 2020). The inoculant’s functionality and persistence are strongly influenced by their complex interactions within the soil microbiota and the environment (Barea et al., 2005; Trivedi et al., 2020; Pozo et al., 2021). Inconsistent or ineffective performance of single strain inoculants can be related to limited competitiveness against indigenous microbes and the varying environmental conditions (Trivedi et al., 2020). It has been proposed that a way to overcome these issues is by combining different strains to cover a wider range of target organisms and conditions (Faust, 2019; Mitter et al., 2019). Yet, successful examples of better performance for microbial consortia are comparatively limited and usually relate to growth or yield promotion (Bradáčová et al., 2019).

Plant microbiome engineering and the design of synthetic microbial communities (SynComs) to improve crop productivity and resilience is a major research topic in this decade (Arif et al., 2020; Liu et al., 2020; Trivedi et al., 2020). SynComs may improve the stability of biocontrol practices as microbial consortia are expected to deal better than single-strain inoculants with the large diversity of environmental challenges encountered in practice (Sarma et al., 2015; Arif et al., 2020; Pozo et al., 2021). Besides acquiring this plasticity, the consortium can combine diverse modes of action, likely providing better pest or disease control than single microorganisms with their specific abilities (Sarma et al., 2015). Yet, most SynComs studies focus exclusively on bacteria, whereas fungi are major biocontrol agents (Pozo et al., 2021). Including fungi in the consortia would likely expand the range of functions and potential colonization niches of these mixed inoculants (Srivastava et al., 2010; Pozo et al., 2021). Thus, combining both bacteria and fungi in SynComs design is expected to result in a multifunctional and more resilient product for biocontrol; this is the basis of this study.

Diving deeper mechanistically, two main groups of biocontrol mechanisms are described: (i) those with direct effects on the attacker and (ii) those with indirect, usually plant-mediated effects. Direct effects are mostly based on microbial antagonism through antibiosis, competition for nutrients or colonization niches, and/or parasitism (Whipps, 2001). Indirect mechanisms reducing pathogen proliferation, aggressiveness, or damage commonly involve plant-mediated effects. Beneficial microorganisms can improve the plant nutritional status, leading to damage compensation and tolerance, and stimulate the plant immune system, priming plant defenses and leading to induced systemic resistance (ISR) to diverse aggressors (Pieterse et al., 2014; Barea, 2015; Pineda et al., 2015; Gruden et al., 2020; De Kesel et al., 2021). Among rhizospheric microorganisms, plant growth-promoting rhizobacteria (PGPR), *Trichoderma* spp., and arbuscular mycorrhizal fungi (AMF) have been shown to effectively protect plants against diverse pests and diseases through different mechanisms (Pozo and Azcón-Aguilar, 2007; Barea et al., 2013; Pieterse et al., 2014; Barea, 2015; Pineda et al., 2015).

Plant growth-promoting rhizobacteria have been shown to control plant pathogens through antibiosis, reduction of pathogen virulence, competition for iron, plant growth promotion, and ISR (Lugtenberg and Kamilova, 2009; Barea, 2015). Most reported PGPR antagonists are from the genera *Bacillus* and *Pseudomonas* (Haas and Défago, 2005; Santoyo et al., 2012).

Regarding fungi, *Trichoderma* spp. is the most widely used BCA in agriculture, and many *Trichoderma*-based products are available in the market (Woo et al., 2014). These fungi are extremely efficient not only for the control of fungal pathogens mainly through direct antagonism but also stimulating plant defenses (Harman et al., 2004; Martínez-Medina et al., 2014; Woo et al., 2014). Finally, AMF is commercialized as biostimulants in agriculture. These obligate biotrophs improve plant nutrient uptake and tolerance/resistance to multiple stresses, being able to protect the host plant against diverse pathogens and pests (Jung et al., 2012; Sanmartín et al., 2020; Rivero et al., 2021). AMF does not produce antibiotics, but compete with the pathogens for nutrients and colonization sites and boosts the defensive capacity of plants, leading to ISR (Pozo and Azcón-Aguilar, 2007; Jung et al., 2012).

In this study, we test the hypothesis that microbial consortia are more versatile than individual microbial inoculants, displaying an extended functionality in the biocontrol of a wider range of plant diseases and application methods through the combination of diverse modes of action. For that, we designed different SynComs by carefully selecting diverse and well-characterized microbial biocontrol agents, including *Bacillus* spp., *Pseudomonas* spp., *Trichoderma* spp., and the AMF *Rhizophagus irregularis* and compared the ability to control root and shoot pathogens when applied individually or in combinations as SynComs. Using different inoculation methods and two agronomically relevant pathosystems (tomato plants challenged with *Fusarium oxysporum* or *Botrytis cinerea* as root and shoot pathogens, respectively), we demonstrate the advantages of targeting microbial consortia as versatile products for efficient biocontrol of diverse plant diseases.

## Materials and Methods

### Microbial Consortia Design

A careful selection of beneficial microorganisms to create synthetic microbial consortia was performed focusing on the main groups of rhizospheric beneficial microorganisms such as PGPR, mycoparasitic fungi, and AMF. An extensive literature review on biocontrol studies of known BCAs was performed, taking also into account as potential candidates the microbial strains available at Koppert Biological Systems. The most relevant studies considered are summarized in Supplementary Table 1.

As a result, we chose two *Bacillus amyloliquefaciens* strains CECT 8238 and CECT 8237, formerly known as *Bacillus subtilis* UMAF6614 and UMAF6639, respectively (Magno-Perez-Bryan et al., 2015), and *Pseudomonas chlororaphis* MA 342 and *Pseudomonas azotoformans* F30A (Abuamsha et al., 2011a; Levenfors et al., 2014). From fungi, we selected *Trichoderma harzianum* strains T22 and ESALQ1306 (Geraldine et al., 2013; Coppola et al., 2019), and for the ISR bioassay, we included additionally the AMF *R. irregularis* MUCL 57021.

### Microbe Growing Conditions and Inoculum Preparation

*Bacillus amyloliquefaciens* strains were grown on tryptone soya agar (TSA, Oxoid, Basingstoke, United Kingdom) for 24 h at 28°C. After that, a single colony from TSA culture was inoculated in 25 ml of Difco sporulation medium (DSM; Nicholson and Setlow, 1990) and incubated for 48 h at 28°C in a rotatory shaker (200 rpm). Spores were quantified using a Bürker-Türk counting chamber, then centrifuged at 5,000 rpm for 15 min, and after discarding the supernatant, the pellet containing the spores was resuspended in sterile tap water to a final concentration of 1 × 10^7^ spores/ml.

*Pseudomonas azotoformans* and *P. chlororaphis* were grown on TSA for 24 h at 28°C. Liquid pre-culture was prepared using tryptone soya broth (TSB, Oxoid, Basingstoke, United Kingdom) inoculated with a single bacterial colony from TSA culture and incubated overnight at 28°C with rotary shaking at 200 rpm. After that, 1 ml of pre-culture was inoculated in 25 ml of TSB medium and placed in a rotatory shaker (200 rpm) at 28°C. After 150 min of incubation, with bacterial growth in exponential phase, the cell concentration was calculated measuring the O.D. (620 nm) of the bacterial culture on Shimadzu UVmini-1240 Spectrophotometer. The bacterial culture was centrifuged at 5,000 rpm for 15 min, and after discarding the supernatant, the pellet containing the bacterial cells was resuspended in sterile tap water to a final concentration of 1 × 10^7^ colony forming unit (cfu)/ml.

*Trichoderma harzianum* strains were cultured on potato dextrose agar (PDA, Difco, Le Pont de Claix, France) for 7 days at room temperature. Spores were collected from sporulating plates in sterile tap water, and the concentration of the spore suspension was quantified using a Bürker-Türk counting chamber and adjusted to 1 × 10^7^ spores/ml.

*Rhizophagus irregularis* was grown in a monoxenic culture on a minimal (M) medium and using *Agrobacterium rhizogenes*-transformed carrot (*Daucus carota*) roots as a host root (St-Arnaud et al., 1996). To extract the AMF spores, citrate buffer 0.01 M (pH = 6) was added to a sporulating AMF culture in a proportion of 3:1 (v/v) and placed in a rotary shaker for 1 h to dissolve the agar. AMF spores were recovered from the solution using sieves of different sizes (250 and 53 μm) and resuspended in sterile tap water at final concentrations of 1,000 spores/ml.

### Pathogenic Fungi, Growing Conditions, and Inoculum Preparation

Two major fungal pathogens causing important crop losses worldwide were tested: *F. oxysporum* f.sp. radicis-lycopersici as soil pathogen and the necrotrophic shoot pathogen *B. cinerea* strain B05.10.

*Fusarium oxysporum* was grown on PDA at 25°C for 4 days. For spore production, 25 plugs of 4 mm diameter with new growing mycelia were removed from the PDA plates and transferred to 500 ml Erlenmeyer containing 200 ml of Czapek Dox Broth (Oxoid, Basingstoke, United Kingdom) and placed in a rotary shaker (110 rpm) at room temperature. After 4 days of incubation, the liquid culture was filtered using a sterile miracloth filter, and the spore concentration was quantified using a Bürker-Türk counting chamber. The resulting spore suspension was centrifuged at 9,500 rpm for 15 min and after discarding the supernatant, the pellet containing the spores was resuspended in sterile tap water to a final concentration of 1 × 10^8^ spores/ml.

*Botrytis cinerea* was cultured on PDA at 20°C. Spores were collected from sporulating 14 days old plates in potato dextrose broth (PDB, Difco, Le Pont de Claix, France), and the concentration of the spore suspension was quantified using a Bürker-Türk counting chamber and adjusted to 1 × 10^6^ spores/ml.

### *In vitro* Antagonism Assay

The antagonistic activity of the individual strains *Bacillus amyloliquefaciens* CECT 8238 and CECT 8237, *P. azotoformans, P. chlororaphis*, and *T. harzianum* T22 and ESALQ1306 were initially evaluated *in vitro*, in confrontation assays against *F. oxysporum* and *B. cinerea*. For *Trichoderma*, one PDA plug (4 mm) of *Trichoderma* culture and one of the pathogen cultures were placed on PDA plates with 4 cm of distance from each other. For *Bacillus* and *Pseudomonas*, 10 μl drop of TSB liquid culture grown overnight was used instead of PDA plugs. As a control, a plug of the pathogen culture was placed in the Petri dish without any antagonist. All treatments were replicated three times. All plates were incubated at 25°C for 7 days. The radius of the pathogen colony in the confrontation plates was measured and compared to the radius of the pathogen colony in the control plates.

### *In planta* Bioassays

Biocontrol potential was tested *in planta* through several bioassays including diverse inoculation methods and targeting different pathogens. This strategy allows testing *in vivo* different modes of action ranging from direct antagonism to indirect plant-mediated effects. Thus, we tested through seed inoculation suppression of the root pathogen *F. oxysporum* and ISR against the foliar pathogen *B. cinerea*, and suppression of *B. cinerea* by foliar spray application.

#### Microbial Treatments

In all bioassays, individual microorganisms and different synthetic consortia were tested (Supplementary Table 2). All microorganisms tested individually were applied at 1 × 10^7^ cfu or spores/plant in the seed application and at 1 × 10^7^ cfu or spores/ml in the foliar application. For the AMF treatments, a suspension of 1,000 spores of *R. irregularis* was applied per plant. Regarding the consortia, the first microbial consortium, SynCom1, was composed of one strain from each genus (*Bacillus amyloliquefaciens* CECT 8238, *P. azotoformans* F30A, *and T. harzianum* T22). The second one, SynCom2, was composed of all selected microorganisms (*Bacillus amyloliquefaciens* CECT 8238 and CECT 8237, *P. azotoformans* F30A, *P. chlororaphis* MA 342, and *T. harzianum* T22 and ESALQ1306). Both consortia were tested at two doses: A—the same amount of each microorganism in both consortia (1 × 10^7^ cfu each, that is a total of 3 × 10^7^ cfu per seed or ml for SynCom1, 6 × 10^7^ cfu per seed or ml for SynCom2) or B—same total cfu per consortia (3.33 × 10^6^ cfu per microorganism in SynCom1 or 1.67 × 10^6^ cfu in SynCom2, for a total of 1 × 10^7^ cfu per seed or ml in both).

#### Substrate, Seed Surface Sterilization, and Plant Growing Conditions

*Solanum lycopersicum* cv. Money maker seeds (Vreeken’s Zaden, Dordrecht, Netherlands) were surface-sterilized by immersion in 5% sodium hypochlorite solution for 1 min followed by at least three washing steps in sterile water for 10 min each. The surface sterilized seeds were dried in a laminar flow cabinet and used for the experiments. The growing substrate was gamma-irradiated nutrient poor peat soil (BVB, Netherlands). All experiments were performed in a growing chamber at Koppert B.V. (Berkel en Rodenrijs, Netherlands) under controlled conditions (25°C:23°C day:night with photoperiod 16 h:8 h light:dark and 60% of relative humidity).

#### Bioassay: Suppression of *Fusarium oxysporum in planta*

Rectangular plastic containers of 18 cm × 13 cm × 6 cm (length × width × height) were filled with 300 g of soil previously moistened with tap water (300 ml/1,000 g of soil) and infected with 1 × 10^6^ conidia/g of soil *F. oxysporum* f.sp. radicis-lycopersici conidia. The *F. oxysporum* conidia were carefully mixed through the soil by hand. Then, 12 seeds were sown in each container in a regular grid and inoculated with the microbial treatments (Supplementary Table 2) by pipetting the microbial suspension to each seed. Finally, the seeds were covered with sterile vermiculite to avoid desiccation and undesired contaminations. We included two control treatments: a “non-diseased control” using the same soil and conditions but without the addition of *F. oxysporum* and microbial treatments, and a “disease control” using the same pathogen-infected soil but without beneficial microbes. Each treatment was replicated five times. We used a randomized complete block design. Each treatment was randomly assigned to each block. Plant survival was evaluated 15 days after sowing by counting the number of healthy tomato plantlets in each container.

#### Bioassay: Suppression of *Botrytis cinerea in planta*

Tomato seeds were sown in pots filled with 250 ml of soil (one seed per pot). Plants were grown for 7 weeks and watered two times per week with water and once per week with Long Ashton nutrient solution (Hewitt, 1966). The individual and the consortia treatments described above (Supplementary Table 2) were applied to one fully developed leaf by spraying its surface until runoff. The disease control treatment was treated similarly, applying the same amount of sterile water but lacking any BCA microbial propagules. Each treatment was replicated six times. Treated leaves were detached after the application, using a scalpel, and used for the bioassay. Each leaflet of the detached leaves was inoculated with one 4 μl drop of *B. cinerea* conidia suspension (1 × 10^6^ conidia/ml). The leaves were placed in six sealed boxes with high humidity at 20°C, locating one replicate from each treatment in each box. About 60 h after infection, the diameter of the resulting necrotic lesions was measured using a digital caliper.

#### Bioassay: Induced Systemic Resistance Against *Botrytis cinerea*

Tomato seeds were sown in pots containing 250 ml of soil (one seed per pot) and the microbial treatments (Supplementary Table 2) were applied by pipetting the microbial suspension to the seeds. In this experiment, the AMF *R. irregularis* was also included, both, individually and in the consortia. A disease control treatment was included where the seeds only received water without any BCA microbial addition. Each treatment was replicated 12 times. We used a randomized complete block design. Plants were watered two times per week with water and once per week with Long Ashton nutrient solution (Hewitt, 1966) but with reduced phosphorous concentration (50% of the standard concentration) to ensure mycorrhizal establishment. After 5 weeks, one fully developed leaf from each plant was detached using a scalpel, and each leaflet was inoculated with one 4 μl drop of *B. cinerea* conidia suspension (1 × 10^6^ conidia/ml). The leaves were placed in 12 sealed boxes with high humidity at 20°C and locating one replicate from each treatment in each box. About 48 h after infection, the diameter of the necrotic lesions was measured using a digital caliper.

#### Bioassay: Strains-Compatibility

Rectangular plastic containers of 18 cm × 13 cm × 6 cm (length × width × height) were filled with 300 g of soil previously moistened with tap water (300 ml/1,000 g of soil). Then, 12 surface-sterilized tomato seeds were sown in each container in a regular grid. The seeds were inoculated with the different microbial treatments (Supplementary Table 2) by pipetting the microbial suspension to each seed. Each microbial strain (except *R. irregularis*) was initially inoculated at 1 × 10^7^ cfu/plant, resulting in a total concentration of 4 × 10^5^ cfu/g of soil for each strain (12 plants/300 g of soil). Finally, the seeds were covered with sterile vermiculite to avoid desiccation and undesired contaminations. We included a control treatment without any microbial inoculation. Each treatment was replicated five times. We used a randomized complete block design. Microbial colonization was evaluated 15 days after sowing using methods described in the next section.

#### Quantification of Microbes and Root Mycorrhizal Colonization

For the different bacteria and *Trichoderma*, we estimated for each genus the number of colony forming units (cfu) per gram of rhizospheric soil. For this, 1 g of rhizospheric soil was sampled, diluted in 9 ml of sterile tap water, and homogenized in a horizontal shaker at 350 rpm for 1 h. Serial dilutions were plated on PDA + igepal (11 ml/L) + tetracycline (50 μg/ml) when targeting *Trichoderma* and on TSA + natamycin (0.1 g/L) when targeting bacteria. The plates were then incubated at 25°C and cfu were counted after 24 h for bacteria and after 48 h for *Trichoderma*. In consortia treatments, *Bacillus* spp., *Pseudomonas* spp., and *Trichoderma* spp. were distinguished morphologically, as they are well-characterized strains in the Koppert collection (Supplementary Figures 1A,B). Microbial identity was confirmed in representative colonies from each type by PCR using specific primers for *Trichoderma, Bacillus*, or *Pseudomonas* spp. For treatments including AMF, mycorrhizal colonization was estimated by ink staining fungal structures within the roots. For that, roots were washed and sampled upon harvesting and cleared in 10% KOH, and the AMF structures were stained with 5% ink in 2% acetic acid (Vierheilig et al., 2005). The percentage of root length colonized by the AMF was quantified using the gridline intersection method (Giovannetti and Mosse, 1980) under a light microscope.

### Statistical Analysis

Data were analyzed using R statistical language, version 4.0.5 (R Core Team, 2021), and figures were produced using the package ggplot2 (Wickham, 2009). The effect of microbial treatments (single strains and synthetic communities) on the necrotic lesions caused by *B. cinerea*, microbial colonization after single and combined inoculations, and the effect of single strains on *B. cinerea* and *F. oxysporum* radial growth was assessed using a general linear model with blocks as an error term and microbial treatments as a fixed effect. To examine whether microbial treatments influenced the probability of the tomato seedlings to survive to the soil pathogenic fungus *F. oxysporum*, a generalized linear model with binomial distribution and logit link function and blocks as an error term was performed. *Post hoc* comparisons among microbial treatments were based on the Tukey honestly significant difference (HSD). Model validation was performed graphically by inspecting the residuals and fitted values (Zuur and Ieno, 2016).

## Results

### The First Step: Consortia Design

Upon a thorough literature review, we selected bacterial and fungal groups/genera with well-documented potential to control plant pathogens, trying to compile diverse mechanisms including antibiosis, competition for iron and other nutrients, and colonization sites, mycoparasitism, and induction of plant resistance. Strains from the selected groups available at the Koppert microbial collection were: *Bacillus amyloliquefaciens* strains CECT 8238 and CECT 8237, *P. chlororaphis* MA 342, and *P. azotoformans* F30A, *T. harzianum* strains T22 and ESALQ1306, and the AMF *R. irregularis* MUCL 57021 (Supplementary Table 1). Two synthetic communities were designed, one combining one strain for each genera (SynCom1), and another in which all selected microbes were included (SynCom2).

### Exploring *in vitro* Antagonistic Activity Against Soil and Leaf Pathogens

As a first screening to move into the biocontrol potential of the selected individual strains, their antagonistic activity was tested in an *in vitro* dual confrontation assay. All selected BCA strains decreased *F. oxysporum* radial growth compared to the control plates (*p* < 0.05; Supplementary Figure 2A). Both *T. harzianum* strains showed the strongest antifungal activity, with about 80% reduction of the pathogen radial growth (*p* < 0.05; Supplementary Figure 2A). Similarly, all individual strains reduced *B. cinerea* radial growth compared to the control, and *T. harzianum* T22 was the most effective strain with a 90% reduction of pathogen growth (*p* < 0.05; Supplementary Figure 2B).

### Assessing the Potential to Directly Suppress Soil Diseases *in planta*

**FIGURE 1:**
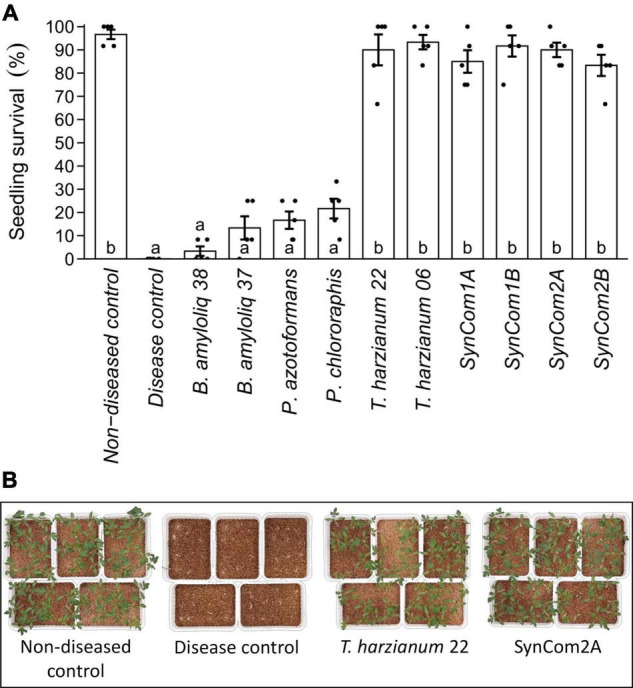
Effect of microbial inoculation on disease caused by the soil-borne pathogen *Fusarium oxysporum.* **(A)** Survival of tomato plants after 15 days of growth in *F. oxysporum*-infected soil. Seeds were either water-inoculated (“disease control”) or inoculated with the individual or consortia treatments (see Supplementary Table 2). A “non-diseased control” was also included, where water-inoculated seeds were sown in soil without *F. oxysporum*. Single strains were inoculated at 1 × 10^7^ cfu/plant and the consortia were inoculated at the same concentration for each microorganism (SynCom1A, SynCom2A) or at 1 × 10^7^ cfu/plant total microbial concentration (SynCom1B, SynCom2B). Bars represent predicted mean ± SE of the probability of seedling survival based on a generalized linear model with binomial distribution and logit link function. Black dots represent raw data points. Treatments not sharing a letter in common are significantly different based on the Tukey honestly significant difference (HSD) test (*p* < 0.05, *n* = 5). **(B)** Survival of plant seedlings in *F. oxysporum*-infected soil. Pictures illustrate plant survival in non-diseased and disease control, *Trichoderma harzianum*, and SynCom2A treatments.

The research was scaled up using a tomato-*Fusarium*-soil system, comparing the biocontrol activity of the individual microbial strains and the differently designed consortia (SynCom1, SynCom2). The pathogen fully compromised plant survival, as no plants survived in the disease control, while almost 100% survival was found in the absence of the pathogen (non-diseased control) (Figure 1A). None of the individual bacterial strains significantly increased plant survival compared to the disease control. In contrast, both *T. harzianum* strains and all of the SynComs were able to efficiently suppress *F. oxysporum*, increasing plant survival above 80% (*p* < 0.05, Figure 1A). In fact, plant survival in the *T. harzianum* and consortia treatments reached the levels of the non-diseased control (*p* < 0.05, Figure 1A). These results not only show the potential of *T. harzianum* but also indirectly the compatibility/tolerance of the other isolates as this high protection level was maintained in the consortia treatments (Figures 1A,B).

### Assessing the Potential to Directly Suppress Foliar Diseases *in planta*

**FIGURE 2:**
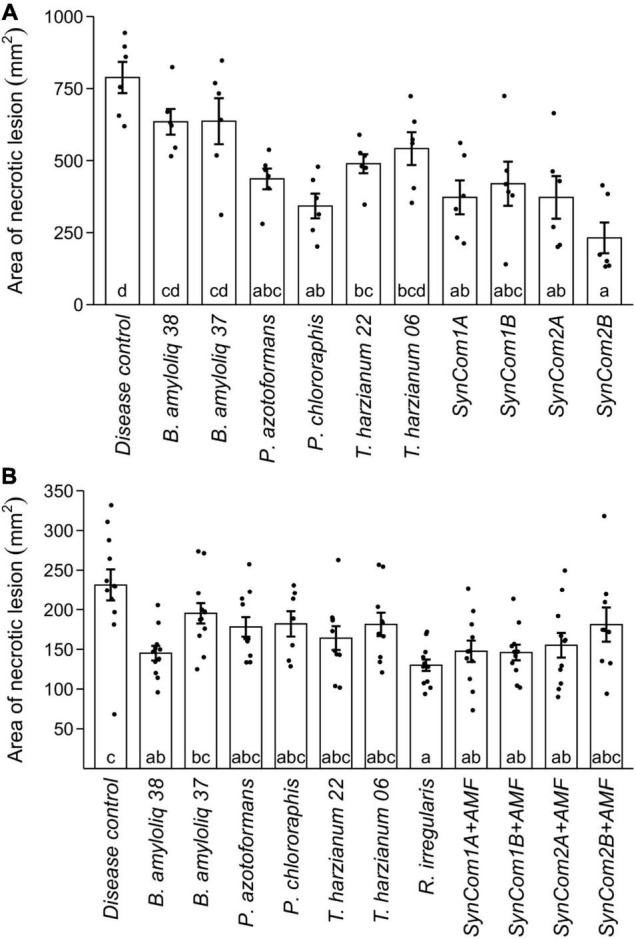
Effect of microbial inoculation on disease caused by the foliar pathogen *Botrytis cinerea.* **(A)** Area of necrotic lesions in plants pre-treated by foliar spray with the different treatments (see Supplementary Table 2). Water-treated plants (no BCA treatment) were included as disease control. **(B)** Area of necrotic lesions in plants inoculated at sowing either with water (disease control) or with the different microbial treatments (see Supplementary Table 2) to determine ISR. Single strains were inoculated at 1 × 10^7^ cfu/ml in **(A)** and cfu/plant in **(B)**, and the consortia were inoculated at the same concentration for each microorganism (SynCom1A, SynCom2A) or at 1 × 10^7^ cfu/ml in **(A)** and cfu/plant in **(B)** as total microbial concentration (SynCom1B, SynCom2B). +AMF indicates consortia co-inoculated with 1,000 spores/plant of *Rhizophagus irregularis.* Bars represent means ± SE and black dots represent raw data. Treatments not sharing a letter in common are significantly different based on the general linear model and Tukey honestly significant difference (HSD) test [*p* < 0.05, *n* = 6 in **(A)**, *n* = 12 in **(B)**].

The antagonistic potential of single strains and consortia against the foliar pathogen *B. cinerea* was also tested *in planta*, applying the BCA treatments by spraying the leaves before *B. cinerea* infection. Among single microbial treatments, *P. chlororaphis*, *P. azotoformans*, and *T. harzianum* T22 were able to reduce the area of the necrotic lesion caused by *B. cinerea* by 56, 45, and 38%, respectively, compared to the control treatment (*p* < 0.05, Figure 2A). Remarkably, all the microbial consortia treatments reduced *B. cinerea* lesion area by about 50% as compared to the disease control, reaching up to a 70% reduction in SynCom2B (*p* < 0.05, Figure 2A). The higher antagonistic effect against *B. cinerea* was therefore achieved by *P. chlororaphis* (56%) and the SynCom2B (Supplementary Figure 3).

### Moving Into Plant-Mediated Control: Inducing Systemic Resistance

In addition to the direct antagonistic effect of the foliar application against *B. cinerea*, we evaluated the capacity of the microbial treatments to activate plant systemic resistance. We tested the potential plant-mediated effects by avoiding direct contact between the BCAs and the pathogen. In this experiment, the AMF *R. irregularis* was included both individually and in the consortia due to the reported capacity of AMF to induce ISR and their current interest as inoculants in agriculture. Among the individual treatments, only *Bacillus amyloliquefaciens* CECT 8238 and *R. irregularis* were able to induce ISR against *B. cinerea*, reducing the area of the necrotic lesions by 38 and 44%, respectively, as compared to the control treatment (*p* < 0.05, Figure 2B). The consortia also achieved significant plant-mediated protection against *B. cinerea*, with SynCom1A, SynCom1B, and SynCom2A reducing lesions by 33–37% as compared to the control (*p* < 0.05, Figure 2B). Again, a similar reduction in disease symptoms was achieved by the consortia and the best performing individual treatments in this pathosystem.

Taking into account all the bioassays performed, SynComs was more versatile than the individual strains, showing effective biocontrol across the different pathosystems and inoculation methods, as summarized in Table 1.

**TABLE 1:** Effects of the microbial treatments tested in the different *in planta* bioassays.

**Microbial treatment**	**Suppression *Fusarium oxysporum***	**Suppression *Botrytis cinerea***	**ISR against *Botrytis cinerea***
*Bacillus amyloliquefaciens* CECT 8238	o	o	+
*Bacillus amyloliquefaciens* CECT 8237	o	o	o
*Pseudomonas azotoformans*	o	+	o
*Pseudomonas chlororaphis*	o	+	o
*Trichoderma harzianum* T22	+	+	o
*Trichoderma harzianum* ESALQ1306	+	o	o
*Rhizophagus irregularis*	nt	nt	+
SynCom1A	+	+	+
SynCom1B	+	+	+
SynCom2A	+	+	+
SynCom2B	+	+	o

*“+” and “o” indicates statistically different effect from the control treatment and no effect, respectively, based on the Tukey honestly significant difference (HSD). “nt” indicates that the microbial treatment was not tested.*

### Microbial Compatibility

The repeated success of the consortia across all the *in planta* bioassays supports the strains compatibility. In fact, the SynComs performance in biocontrol was not significantly different from that achieved by the best performing BCA strain in any of the experiments. We further investigated the compatibility of the components in a new experiment aiming to compare the colonization of each microorganism in the single or SynCom treatments after interacting in the tomato rhizosphere for 15 days. The absence in the soil of indigenous species from any of the inoculated genera (*Bacillus*, *Pseudomonas*, and *Trichoderma*) was confirmed in the control treatment plates (Supplementary Figure 1A). Each microbial strain (except *R. irregularis*) was initially inoculated at a total concentration of 4 × 10^5^ cfu/g of soil for each strain (both in the individual microbial treatments and in consortia).

**FIGURE 3:**
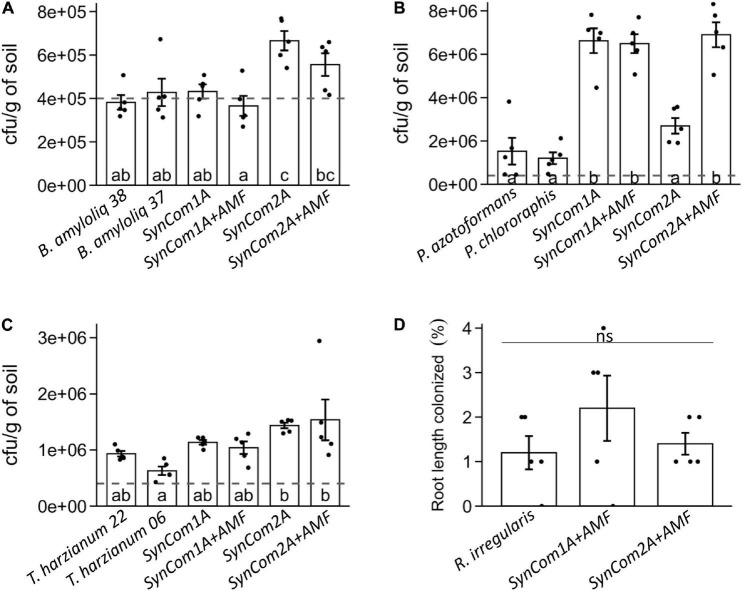
Rhizospheric soil colonization by **(A)** *Bacillus* spp., **(B)** *Pseudomonas* spp., and **(C)** *Trichoderma* spp., expressed as cfu/g of soil, and **(D)** mycorrhizal colonization by *Rhizophagus irregularis* represented as the percentage of root length colonized by the fungus. Plants were inoculated at sowing with the individual or consortia treatments (see Supplementary Table 2) and grown for 15 days. +AMF indicates consortia co-inoculated with 1,000 spores/plant of *R. irregularis.* Bars represent mean ± SE. Dashed lines represent the initial concentration inoculated for each microorganism (4 × 10^5^ cfu/g of soil). Treatments not sharing a letter in common are significantly different based on the general linear model and Tukey honestly significant difference (HSD) test (*p* < 0.05, *n* = 5).

*Bacillus* spp. abundance at the end of the experiment was similar to that initially inoculated both in single strain and SynCom1 treatments (Figure 3A). In SynCom2 treatments, both *Bacillus* strains were co-inoculated, and the abundance of *Bacillus* spp. in the soil was even higher, around 6 × 10^5^ cfu/g of soil (Figure 3A). These results confirm the successful establishment of both *Bacillus amyloliquefaciens* strains, both when inoculated individually and in consortia.

In the single strain treatments, *Pseudomonas* spp. abundance increased compared to the initial inoculation (up to 1.5 × 10^6^ and 1.2 × 10^6^ cfu/g of soil in *P. azotoformans* and *P. chlororaphis*, respectively) (Figure 3B), evidencing the good colonization ability of *Pseudomonas* spp. Remarkably, *Pseudomonas* spp. abundance in soil increased more than four times in SynCom1 (containing *P. azotoformans*) compared to the individual *P. azotoformans* treatment (around 6.5 × 10^6^ cfu/g of soil) (Figure 3B). Regarding SynCom2 treatments in the absence of AMF *Pseudomonas* spp., abundance was 2.7 × 10^6^ cfu/g of soil, corresponding to the sum of both inoculated *Pseudomonas* species in SynCom2, whereas in SynCom2 + AMF, their abundance was more than double (6.9 × 10^6^ cfu/g of soil), pointing to a potential positive effect of AMF presence in this consortium (Figure 3B).

*Trichoderma* spp. abundance in the individual treatments was 9.3 × 10^5^ and 6.3 × 10^5^ cfu/g of soil in *T. harzianum* T22 and ESALQ1306, respectively, which in the case of T22 is more than double of the concentration inoculated (Figure 3C). Regarding the consortia, *Trichoderma* spp. abundance was in a similar range than the individual inoculations: 1 × 10^6^ cfu/g of soil in SynCom1 (where only T22 was present) and around 1.5 × 10^6^ cfu/g of soil in SynCom2 treatments equivalent to the sum of both *Trichoderma* strains co-inoculated in this consortium (Figure 3C).

Finally, the percentage of root length colonized by *R. irregularis* was 1.2% when applied individually (Figure 3D). Root colonization was similar in both consortia treatments, (SynCom1 + AMF and SynCom2 + AMF) (Figure 3D), confirming that mycorrhizal colonization was not significantly affected when inoculated in consortia. The low percentages are common in the early stages of the colonization (only 2 weeks upon AMF inoculation). To compare the treatments in more advanced stages of the mycorrhizal symbiosis, mycorrhizal colonization was quantified in the roots of the ISR bioassay, corresponding to plants growing with the AMF for 5 weeks. Mycorrhizal colonization reached 40% in the individual treatment, and these levels remained unaltered in both SynCom1 and SynCom2 treatments at any of the tested doses (Supplementary Figure 4).

## Discussion

In the present study, by combining well-characterized and compatible microorganisms, including bacteria and fungi, we demonstrated the potential of microbial consortia to effectively control fungal pathogens with different lifestyles through direct and plant-mediated disease suppression and using different application methods. Our findings pinpoint the design of synthetic microbial consortia for biocontrol of plant pathogens as a potential strategy to extend the functionality and versatility of microbial biological control.

### A Dilemma to Face

**FIGURE 4:**
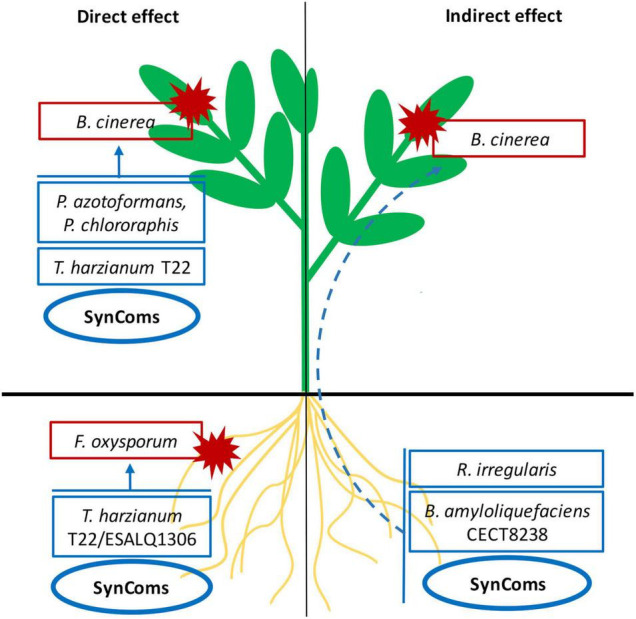
Summary of the microbial treatments showing suppressive effects on *Botrytis cinerea* and *Fusarium oxysporum* through direct antagonism (arrows) or the induction of plant of systemic resistance (dashed arrow) after foliar spray and seed application.

Across the different experiments, different individual microorganisms were the most effective in the different scenarios, depending on the type of pathogen or the strategy used for its control. Remarkably, the consortia effectively controlled all pathogens in all different bioassays, both through direct antagonism by seed or foliar application, or inducing plant systemic resistance against foliar pathogens by seed inoculation (results summarized in Figure 4). The bioprotection achieved by the consortia was always similar to that of the best performing single strains. Although no significant synergism was detected, no negative interactions were observed, in contrast to some studies reporting positive and negative effects by the combination of BCAs (Freeman et al., 2004; Abo-Elyousr et al., 2009; Elliott et al., 2009; Ruano Rosa et al., 2014). Our results illustrate the topical dilemma of selecting single beneficial microbes versus SynComs for biological control. Strictly from the potential efficacy point of view, SynComs offered the widest protection after comparing the single components and several consortia across the soil and foliar threats and through direct and indirect actions. Yet, the efficacy was not higher than that of the best performing single strain and, in most cases, more than one individual microbe provided effective control. Considering the current high costs and outstanding long process for registering microbial products, targeting single strain or SynCom products is a tough dilemma to face from the commercial point of view. Nevertheless, the advantage of SynComs as a more versatile tool may become more apparent under field conditions, considering the variability of growing conditions and the uncertainty of the potential challenges to be faced—what pathogens or pests would be threatening the crop. We postulate that in the field, under commercial conditions, the benefits for the SynComs would further differentiate to the individual components. Thorough validation of results in field conditions will give the answer.

### Selecting a Potentially Powerful Pool as Step One

For the design of the synthetic microbial consortia, we selected different strains aiming to combine different mechanisms for biocontrol from the production of diverse antimicrobial metabolites through mycoparasitism to ISR. *Bacillus amyloliquefaciens* strains CECT 8238 and CECT 8237 have been shown to promote plant growth and effectively control diverse microbial pathogens through direct antagonism or indirectly through ISR (Romero et al., 2007; García-Gutiérrez et al., 2012, 2013; Magno-Perez-Bryan et al., 2015). *P. chlororaphis* MA342 has been described to effectively control seed and soil pathogens *via* direct antagonism (Tombolini et al., 1999; Abuamsha et al., 2011a) and protecting against leaf pathogens through seed priming (Abuamsha et al., 2011b). *P. azotoformans* F30A effectively enhance plant emergency and growth (Levenfors et al., 2014) and can also induce ISR to leaf pathogens (Sang et al., 2014; Bouaoud et al., 2018). *T. harzianum* strain T22 is one of the best characterized and commercialized *Trichoderma* strains. It effectively antagonizes soil pathogens (Wilson et al., 2008; Percival et al., 2011; Roberti et al., 2015; Fatouros et al., 2018) and can trigger ISR against diverse above- and belowground attackers (Tucci et al., 2011; Vitti et al., 2016; Coppola et al., 2017, 2019; Debode et al., 2018; Pocurull et al., 2020; Alınç et al., 2021). Besides promoting plant growth, *T. harzianum* ESALQ1306 has been shown to highly reduce *Sclerotinia sclerotiorum* disease severity through parasitism and to induce ISR against spider mites (Geraldine et al., 2013; De Oliveira et al., 2018; Barroso et al., 2019; Canassa et al., 2020). In contrast, *R. irregularis* is not a direct antagonist of plant pathogens, but is able to induce ISR against root and foliar pathogens (Martínez-Medina et al., 2011; Sanchez-Bel et al., 2016; Bidellaoui et al., 2019; Campo et al., 2020; Sanmartín et al., 2020). All in all, we selected a potentially powerful pool of microbes, already well characterized in multiple aspects. A number of them are being already exploited commercially either under development into microbial products, or, like *T. harzianum*, already commercialized as BCA by Koppert Biological Systems all over the world from vegetable and ornamental to field and row crops.

### Single Strains Versus SynComs, Variable Outcomes so Far

Most studies focusing on the use of microbial consortia for disease control are looking for synergistic or additive effects, aiming to achieve a higher pest or disease control than their components. While some of these studies have indeed reported positive effects (Guetsky et al., 2001, 2002; Srivastava et al., 2010; Singh et al., 2013; Ruano Rosa et al., 2014; Sylla et al., 2015), many others showed similarly or even less effective in disease control when applying consortia as compared to the application of the individual microbes (Freeman et al., 2004; Abo-Elyousr et al., 2009; Elliott et al., 2009; Ruano Rosa and López Herrera, 2009). However, most of these studies focused on one model system. In contrast, we intended to extend the scope by including an array of target diseases—soil and foliar—and possible mechanisms—direct and indirect control *via* ISR. The SynComs performed consistently well across the different pathosystems. Yet, differences between the SynComs and the individual components were relatively mild in terms of efficacy/degree of control.

### Exploring the Compatibility of the Components of the SynComs

Microbial compatibility is a key factor when designing a microbial consortium, essential for the successful establishment and functionality of the included microorganisms and the success of SynCom products. In our study, the conservation of the biocontrol effectiveness in the SynComs to the same levels as the best performing individual isolates supported the compatibility between the coexisting microorganisms. We further tested their compatibility in our consortia by assessing the microbial survival in a plant–soil-based experiment, and we did not find any negative interaction between them. Instead, *Bacillus* and *Trichoderma* performed in the consortia as good as when individually inoculated, and *Pseudomonas* even benefited from the combination with the other organisms, as they performed better in the SynComs than when inoculated alone. It is important to note that *R. irregularis* was neither negatively affected in early nor late symbiosis stages by the presence of *Trichoderma* spp., as demonstrated by the similar mycorrhizal colonization in roots inoculated with the AMF alone or as part of the consortia. This is remarkable, as the compatibility of *Trichoderma* spp. with mycorrhizal fungi is frequently questioned because of the high mycoparasitic potential of these biocontrol fungi. In fact, *Trichoderma* is able to parasite AMF *in vitro* (Rousseau et al., 1996), but other studies proved their compatibility under more realistic scenarios (i.e., rhizospheric soil) as observed here (Martínez-Medina et al., 2011). Even more, *Trichoderma*-AMF synergistic effects have been reported (Poveda et al., 2019). Although microbe compatibility remains poorly studied, understanding the compatibility between groups or key BCA genera is required for informed decisions in the selection of suitable candidates for SynComs development in biocontrol programs in agriculture.

Overall, our findings highlight the potential multifunctionality of SynComs for biological control. Combining compatible beneficial microorganisms with complementary effects on different targets, direct and indirect mechanisms of control and/or effective under different conditions will lead to the development of biocontrol products with increased versatility. To became commercial products, consistency of the outcomes needs to be tested and finally validated across multiple field trials in the geographical regions where is aimed to be used. This is a key step for the successful application of this sustainable technology in agriculture.

## Data Availability Statement

The original contributions presented in the study are included in the article/Supplementary Material, further inquiries can be directed to the corresponding author.

## Author Contributions

MP, RS, OK, and ZM designed the experiments. ZM performed all the experimental work. OK and ZM analyzed the data. RS and OK critically revised the manuscript. MP and ZM wrote the manuscript. All authors contributed to the article and approved the submitted version.

## Conflict of Interest

The authors declare that the research was conducted in the absence of any commercial or financial relationships that could be construed as a potential conflict of interest.

## Publisher’s Note

All claims expressed in this article are solely those of the authors and do not necessarily represent those of their affiliated organizations, or those of the publisher, the editors and the reviewers. Any product that may be evaluated in this article, or claim that may be made by its manufacturer, is not guaranteed or endorsed by the publisher.

## References

[B1] Ab Rahman S. F. S. Singh E. Pieterse C. M. J. Schenk P. M. (2018). Emerging microbial biocontrol strategies for plant pathogens. *Plant Sci.* 267 102–111. 10.1016/j.plantsci.2017.11.012 29362088

[B2] Abo-Elyousr K. A. M. Hashem M. Ali E. H. (2009). Integrated control of cotton root rot disease by mixing fungal biocontrol agents and resistance inducers. *Crop Protect.* 28 295–301. 10.1016/j.cropro.2008.11.004

[B3] Abuamsha R. Salman M. Ehlers R. U. (2011a). Differential resistance of oilseed rape cultivars (*Brassica napus* ssp. *oleifera*) to *Verticillium longisporum* infection is affected by rhizosphere colonisation with antagonistic bacteria, *Serratia plymuthica* and *Pseudomonas chlororaphis*. *BioControl* 56 101–112. 10.1007/s10526-010-9308-8

[B4] Abuamsha R. Salman M. Ehlers R. U. (2011b). Effect of seed priming with *Serratia plymuthica* and *Pseudomonas chlororaphis* to control *Leptosphaeria maculans* in different oilseed rape cultivars. *Eur. J. Plant Pathol.* 130 287–295. 10.1007/s10658-011-9753-y

[B5] Alınç T. Cusumano A. Peri E. Torta L. Colazza S. (2021). *Trichoderma harzianum* strain t22 modulates direct defense of tomato plants in response to *Nezara viridula* feeding activity. *J. Chemical Ecol.* 1 1260–1263. 10.1007/s10886-021-01260-3 33713251 PMC8116274

[B6] Arif I. Batool M. Schenk P. M. (2020). Plant microbiome engineering: expected benefits for improved crop growth and resilience. *Trends Biotechnol.* 38 1385–1396. 10.1016/j.tibtech.2020.04.015 32451122

[B7] Bakker P. A. H. M. Pieterse C. M. J. de Jonge R. Berendsen R. L. (2018). The Soil-Borne Legacy. *Cell* 172 1178–1180. 10.1016/j.cell.2018.02.024 29522740

[B8] Barea J. M. (2015). Future challenges and perspectives for applying microbial biotechnology in sustainable agriculture based on a better understanding of plant-microbiome interactions. *J. Soil Sci. Plant Nutrit.* 15 261–282. 10.4067/S0718-95162015005000021 27315006

[B9] Barea J. M. Pozo M. J. Azcón R. Azcón-Aguilar C. (2005). Microbial co-operation in the rhizosphere. *J. Exp. Bot.* 56 1761–1778. 10.1093/jxb/eri197 15911555

[B10] Barea J.-M. Pozo M.-J. Azcón R. Azcón-Aguilar C. (2013). “Microbial interactions in the rhizosphere,” in *Molecular microbial ecology of the rhizosphere*, ed. Dehority B. (Hoboken, NJ: John Wiley and Son), 29–44. 10.1051/rnd:19970701

[B11] Barroso F. M. Muniz P. H. P. C. Milan M. D. Santos W. S. dos, Ferreira N. C. (2019). Growth promotion of parsley (*Petroselinum crispum* L.) using commercial strains of *Trichoderma* spp. *J. Agricult. Sci.* 11:493. 10.5539/jas.v11n4p493

[B12] Bidellaoui B. Segarra G. Hakkou A. Trillas M. I. (2019). Beneficial effects of *Rhizophagus irregularis* and *Trichoderma asperellum* strain T34 on growth and fusarium wilt in tomato plants. *J. Plant Pathol.* 101 121–127. 10.1007/s42161-018-0159-y

[B13] Bouaoud Y. Troulet C. Foughalia A. Berge O. Aissat K. Bardin M. (2018). A multi-criteria approach for the selection of efficient biocontrol agents against *Botrytis cinerea* on tomato in Algeria. *BioControl* 63 299–311. 10.1007/s10526-017-9851-7

[B14] Bradáčová K. Florea A. S. Bar-Tal A. Minz D. Yermiyahu U. Shawahna R. (2019). Microbial consortia versus single-strain inoculants: an advantage in PGPM-assisted tomato production? *Agronomy* 9 1–23. 10.3390/agronomy9020105

[B15] Campo S. Martín-Cardoso H. Olivé M. Pla E. Catala-Forner M. Martínez-Eixarch M. (2020). Effect of root colonization by arbuscular mycorrhizal fungi on growth, productivity and blast resistance in rice. *Rice* 13:42. 10.1186/s12284-020-00402-7 32572623 PMC7310045

[B16] Canassa F. D’Alessandro C. P. Sousa S. B. Demétrio C. G. B. Meyling N. V. Klingen I. (2020). Fungal isolate and crop cultivar influence the beneficial effects of root inoculation with entomopathogenic fungi in strawberry. *Pest Manage. Sci.* 76 1472–1482. 10.1002/ps.5662 31659843

[B17] Compant S. Samad A. Faist H. Sessitsch A. (2019). A review on the plant microbiome: Ecology, functions, and emerging trends in microbial application. *J. Adv. Res.* 19 29–37. 10.1016/j.jare.2019.03.004 31341667 PMC6630030

[B18] Coppola M. Cascone P. Chiusano M. L. Colantuono C. Lorito M. Pennacchio F. (2017). *Trichoderma harzianum* enhances tomato indirect defense against aphids. *Insect Sci.* 00 1–9. 10.1111/1744-7917.12475 28475289

[B19] Coppola M. Diretto G. Digilio M. C. Lorito M. Rao R. (2019). Transcriptome and metabolome reprogramming in tomato plants by *Trichoderma harzianum* strain T22 primes and enhances defense responses against aphids. *Front. Physiol.* 10:1–21. 10.3389/fphys.2019.00745 31293434 PMC6599157

[B20] De Kesel J. Conrath U. Flors V. Luna E. Mageroy M. H. Mauch-Mani B. (2021). The induced resistance lexicon: do’s and don’ts. *Trends Plant Sci.* 2021 685–691. 10.1016/j.tplants.2021.01.001 33531282

[B21] De Oliveira J. B. Muniz P. H. P. C. Peixoto G. H. S. De Oliveira T. A. S. Duarte E. A. A. Rodrigues F. (2018). Promotion of seedling growth and production of wheat by using *Trichoderma* spp. *J. Agricult. Sci.* 10:267. 10.5539/jas.v10n8p267

[B22] Debode J. Tender C. De, Cremelie P. Lee A. S. Kyndt T. (2018). *Trichoderma* -inoculated miscanthus straw can replace peat in strawberry cultivation, with beneficial effects on disease control. *Front. Plant Sci.* 9:1–15. 10.3389/fpls.2018.00213 29515613 PMC5826379

[B23] Elliott M. Shamoun S. F. Sumampong G. James D. Masri S. Varga A. (2009). Evaluation of several commercial biocontrol products on European and North American populations of *Phytophthora ramorum*. *Biocontrol Sci. Technol.* 19 1007–1021. 10.1080/09583150903243870

[B24] Fatouros G. Gkizi D. Fragkogeorgi G. A. Paplomatas E. J. Tjamos S. E. (2018). Biological control of *Pythium, Rhizoctonia* and *Sclerotinia* in lettuce: association of the plant protective activity of the bacterium *Paenibacillus alvei* K165 with the induction of systemic resistance. *Plant Pathol.* 67 418–425. 10.1111/ppa.12747

[B25] Faust K. (2019). Microbial consortium design benefits from metabolic modeling. *Trends Biotechnol.* 37 123–125. 10.1016/j.tibtech.2018.11.004 30477738

[B26] Freeman S. Minz D. Kolesnik I. Barbul O. Zveibil A. Maymon M. (2004). Trichoderma biocontrol of *Colletotrichum acutatum* and *Botrytis cinerea* and survival in strawberry. *Eur. J. Plant Pathol.* 110 361–370. 10.1023/B:EJPP.0000021057.93305.d9

[B27] García-Gutiérrez L. Romero D. Zeriouh H. Cazorla F. M. Torés J. A. de Vicente A. (2012). Isolation and selection of plant growth-promoting rhizobacteria as inducers of systemic resistance in melon. *Plant Soil* 358 201–212. 10.1007/s11104-012-1173-z

[B28] García-Gutiérrez L. Zeriouh H. Romero D. Cubero J. de Vicente A. Pérez-García A. (2013). The antagonistic strain *Bacillus subtilis* UMAF6639 also confers protection to melon plants against cucurbit powdery mildew by activation of jasmonate- and salicylic acid-dependent defence responses. *Microb. Biotechnol.* 6 264–274. 10.1111/1751-7915.12028 23302493 PMC3815921

[B29] Geraldine A. M. Lopes F. A. C. Carvalho D. D. C. Barbosa E. T. Rodrigues A. R. Brandão R. S. (2013). Cell wall-degrading enzymes and parasitism of sclerotia are key factors on field biocontrol of white mold by *Trichoderma* spp. *Biol. Control* 67 308–316. 10.1016/j.biocontrol.2013.09.013

[B30] Giovannetti M. Mosse B. (1980). Giovannetti and mosse 1980.pdf. *New Phytol.* 84 489–500.

[B31] Gruden K. Lidoy J. Petek M. Podpeèan V. Flors V. Papadopoulou K. K. (2020). Ménage à trois: unraveling the mechanisms regulating plant–microbe–arthropod interactions. *Trends Plant Sci.* 25 1215–1226. 10.1016/j.tplants.2020.07.008 32828689

[B32] Guetsky R. Shtienberg D. Elad Y. Dinoor A. (2001). Combining biocontrol agents to reduce the variability of biological control. *Biol. Control* 91 621–627. 10.1094/PHYTO.2001.91.7.621 18942990

[B33] Guetsky R. Shtienberg D. Elad Y. Fischer E. Dinoor A. (2002). Improving biological control by combining biocontrol agents each with several mechanisms of disease suppression. *Biol. Control* 92 976–985. 10.1094/PHYTO.2002.92.9.976 18944023

[B34] Haas D. Défago G. (2005). Biological control of soil-borne pathogens by fluorescent pseudomonads. *Nat. Rev. Microbiol.* 3 307–319. 10.1038/nrmicro1129 15759041

[B35] Harman G. E. Howell C. R. Viterbo A. Chet I. Lorito M. (2004). *Trichoderma* species - Opportunistic, avirulent plant symbionts. *Nat. Rev. Microbiol.* 2 43–56. 10.1038/nrmicro797 15035008

[B36] Hewitt E. J. (1966). *Sand and water culture methods used in the study of plant nutrition*, 2nd Edn. Farnham Royal: Commonwealth Agricultural Bureaux, 10.1017/S0014479700021852

[B37] Jung S. C. Martinez-Medina A. Lopez-Raez J. A. Pozo M. J. (2012). Mycorrhiza-induced resistance and priming of plant defenses. *J. Chem. Ecol.* 38 651–664. 10.1007/s10886-012-0134-6 22623151

[B38] Levenfors J. Welch C. F. Fatehi J. Wikstrom M. Rasmussen S. Hökeberg M. (2014). *Fluorescent pseudomonad of the species Pseudomonas azotoformans* for enhancement of plant emergence and growth. Geneva: World Intellectual Property Organization.

[B39] Liu H. Brettell L. E. Qiu Z. Singh B. K. (2020). Microbiome-mediated stress resistance in plants. *Trends Plant Sci.* 25 733–743. 10.1016/j.tplants.2020.03.014 32345569

[B40] Lugtenberg B. Kamilova F. (2009). Plant-growth-promoting rhizobacteria. *Annu. Rev. Microbiol.* 63 541–556. 10.1146/annurev.micro.62.081307.162918 19575558

[B41] Magno-Perez-Bryan M. C. Martinez-Garcia P. M. Hierrezuelo J. Rodriguez-Palenzuela P. Arrebola E. Ramos C. (2015). Comparative genomics within the bacillus genus reveal the singularities of two robust *Bacillus amyloliquefaciens* biocontrol strains. *Mol. Plant Microbe Interact.* 28 1102–1116. 10.1094/MPMI-02-15-0023-R 26035127

[B42] Martínez-Medina A. Alguacil M. del M. Pascual J. A. Van Wees S. C. M. (2014). Phytohormone profiles induced by *Trichoderma* isolates correspond with their biocontrol and plant growth-promoting activity on melon plants. *J. Chem. Ecol.* 40 804–815. 10.1007/s10886-014-0478-1 25023078

[B43] Martínez-Medina A. Roldán A. Albacete A. Pascual J. A. (2011). The interaction with arbuscular mycorrhizal fungi or *Trichoderma harzianum* alters the shoot hormonal profile in melon plants. *Phytochemistry* 72 223–229. 10.1016/j.phytochem.2010.11.008 21145086

[B44] Mitter B. Brader G. Pfaffenbichler N. Sessitsch A. (2019). Next generation microbiome applications for crop production - limitations and the need of knowledge-based solutions. *Curr. Opin. Microbiol.* 49 59–65. 10.1016/j.mib.2019.10.006 31731227

[B45] Nicholson W. L. Setlow P. (1990). “Sporulation, germination and outgrowth,” in *Molecular biological methods for Bacillus*, eds Harwood C. R. Cutting S. M. (New York, NY: John Wiley and Sons), 391–450.

[B46] Percival G. C. Smiley E. T. Fox R. T. V. (2011). Root collar excavation with *Trichoderma* inoculations as a potential management strategy for honey fungus (*Armillaria mellea*). *Arboricult. J.* 33 267–280.

[B47] Pieterse C. M. J. Zamioudis C. Berendsen R. L. Weller D. M. Van Wees S. C. M. Bakker P. A. H. M. (2014). Induced systemic resistance by beneficial microbes. *Annu. Rev. Phytopathol.* 52 347–375. 10.1146/annurev-phyto-082712-102340 24906124

[B48] Pineda A. Soler R. Pozo M. J. Rasmann S. Turlings T. C. J. (2015). Above-belowground interactions involving plants, microbes and insects. *Front. Plant Sci.* 6:1–3. 10.3389/fpls.2015.00318 26074927 PMC4444737

[B49] Pocurull M. Fullana A. M. Ferro M. Valero P. Escudero N. Saus E. (2020). Commercial formulates of *Trichoderma* induce systemic plant resistance to *Meloidogyne incognita* in tomato and the effect is additive to that of the Mi-1 . 2 resistance gene. *Front. Microbiol.* 10:1–10. 10.3389/fmicb.2019.03042 32076417 PMC7006539

[B50] Poveda J. Hermosa R. Monte E. Nicolás C. (2019). *Trichoderma harzianum* favours the access of arbuscular mycorrhizal fungi to non-host Brassicaceae roots and increases plant productivity. *Sci. Rep.* 9 1–11. 10.1038/s41598-019-48269-z 31406170 PMC6690897

[B51] Pozo M. J. Azcón-Aguilar C. (2007). Unraveling mycorrhiza-induced resistance. *Curr. Opin. Plant Biol.* 10 393–398. 10.1016/j.pbi.2007.05.004 17658291

[B52] Pozo M. J. Zabalgogeazcoa I. Vazquez, de Aldana B. R. Martinez-Medina A. (2021). Untapping the potential of plant mycobiomes for applications in agriculture. *Curr. Opin. Plant Biol.* 60:102034. 10.1016/j.pbi.2021.102034 33827007

[B53] R Core Team (2021). *R: A language and environment for statistical computing.* Vienna: R Foundation for Statistical Computing.

[B54] Rändler-Kleine M. Wolfgang A. Dietel K. Junge H. Cernava T. Berg G. (2020). “How microbiome approaches can assist industrial development of biological control products,” in *Integrative Biological Control*, Vol. 20 eds Gao Y. Hokkanen H. Menzler-Hokkanen I. (Berlin: Springer), 201–215. 10.1007/978-3-030-44838-7_13

[B55] Rivero J. Lidoy J. Llopis-Giménez Á Herrero S. Flors V. Pozo M. J. (2021). Mycorrhizal symbiosis primes the accumulation of antiherbivore compounds and enhances herbivore mortality in tomato. *J. Exp. Bot.* 72 5038–5050. 10.1093/jxb/erab171 33884424 PMC8219033

[B56] Roberti R. Bergonzoni F. Finestrelli A. Leonardi P. (2015). Biocontrol of *Rhizoctonia solani* disease and biostimulant effect by microbial products on bean plants. *Microbiol. Ital.* 44 49–61. 10.6092/issn.2465-311X/5742

[B57] Romero D. De Vicente A. Zeriouh H. Cazorla F. M. Fernández-Ortuño D. Torés J. A. (2007). Evaluation of biological control agents for managing cucurbit powdery mildew on greenhouse-grown melon. *Plant Pathol.* 56 976–986. 10.1111/j.1365-3059.2007.01684.x

[B58] Rousseau A. Benhamou N. Chet I. Piché Y. (1996). Mycoparasitism of the extramatrical phase of *Glomus intraradices* by *Trichoderma harzianum*. *Biochem. Cell Biol.* 86 434–443.

[B59] Ruano Rosa D. López Herrera C. J. (2009). Evaluation of *Trichoderma* spp. as biocontrol agents against avocado white root rot. *Biol. Control* 51 66–71. 10.1016/j.biocontrol.2009.05.005

[B60] Ruano Rosa D. Martín Pérez R. Bonilla N. Cazorla F. M. De Vicente A. López Herrera C. J. (2014). Biological control of avocado white root rot with combined applications of *Trichoderma* spp. and rhizobacteria. *Eur. J. Plant Pathol.* 138 751–762. 10.1007/s10658-013-0347-8

[B61] Sanchez-Bel P. Troncho P. Gamir J. Pozo M. J. Camañes G. Cerezo M. (2016). The nitrogen availability interferes with mycorrhiza-induced resistance against *Botrytis cinerea* in tomato. *Front. Microbiol.* 7:1–16. 10.3389/fmicb.2016.01598 27790197 PMC5064179

[B62] Sang M. K. Kim E. N. Han G. D. Kwack M. S. Jeun Y. C. Kim K. D. (2014). Priming-mediated systemic resistance in cucumber induced by *Pseudomonas azotoformans* GC-B19 and *Paenibacillus elgii* MM-B22 against *Colletotrichum orbiculare*. *Phytopathology* 104 834–842. 10.1094/PHYTO-11-13-0305-R 24502209

[B63] Sanmartín N. Pastor V. Pastor-Fernández J. Flors V. Pozo M. J. Sánchez-Bel P. (2020). Role and mechanisms of callose priming in mycorrhiza-induced resistance. *J. Exp. Bot.* 71 2769–2781. 10.1093/jxb/eraa030 31985797 PMC7210776

[B64] Santoyo G. Orozco-Mosqueda M. C. Govindappa M. Santoyo G. Orozco-mosqueda M. C. Govindappa M. (2012). Mechanisms of biocontrol and plant growth- promoting activity in soil bacterial species of *Bacillus* and *Pseudomonas*: a review. *Biocontr. Sci. Technol.* 22 855–872. 10.1080/09583157.2012.694413

[B65] Sarma B. K. Yadav S. K. Singh S. Singh H. B. (2015). Microbial consortium-mediated plant defense against phytopathogens: readdressing for enhancing efficacy. *Soil Biol. Biochem.* 87 25–33. 10.1016/j.soilbio.2015.04.001

[B66] Singh A. Sarma B. K. Upadhyay R. S. Singh H. B. (2013). Compatible rhizosphere microbes mediated alleviation of biotic stress in chickpea through enhanced antioxidant and phenylpropanoid activities. *Microbiol. Res.* 168 33–40. 10.1016/j.micres.2012.07.001 22857806

[B67] Srivastava R. Khalid A. Singh U. S. Sharma A. K. (2010). Evaluation of arbuscular mycorrhizal fungus, fluorescent *Pseudomonas* and *Trichoderma harzianum* formulation against *Fusarium oxysporum* f. sp. *lycopersici* for the management of tomato wilt. *Biol. Control* 53 24–31. 10.1016/j.biocontrol.2009.11.012

[B68] St-Arnaud M. Hamel C. Vimard B. Caron M. Fortin J. A. (1996). Enhanced hyphal growth and spore production of the arbuscular mycorrhizal fungus *Glomus intraradices* in an in vitro system in the absence of host roots. *Mycol. Res.* 100 328–332. 10.1016/S0953-7562(96)80164-X

[B69] Sylla J. Alsanius B. W. Krüger E. Wohanka W. (2015). Control of *Botrytis cinerea* in strawberries by biological control agents applied as single or combined treatments. *Eur. J. Plant Pathol.* 143 461–471. 10.1007/s10658-015-0698-4

[B70] Tombolini R. Van Der Gaag D. J. Gerhardson B. Jansson J. K. (1999). Colonization pattern of the biocontrol strain *Pseudomonas chlororaphis* MA 342 on barley seeds visualized by using green fluorescent protein. *Appl. Environ. Microbiol.* 65 3674–3680. 10.1128/aem.65.8.3674-3680.1999 10427065 PMC91550

[B71] Trivedi P. Leach J. E. Tringe S. G. Sa T. Singh B. K. (2020). Plant–microbiome interactions: from community assembly to plant health. *Nat. Rev. Microbiol.* 18 607–621. 10.1038/s41579-020-0412-1 32788714

[B72] Trivedi P. Schenk P. M. Wallenstein M. D. Singh B. K. (2017). Tiny Microbes, Big Yields: enhancing food crop production with biological solutions. *Microb. Biotechnol.* 10 999–1003. 10.1111/1751-7915.12804 28840959 PMC5609239

[B73] Tucci M. Ruocco M. Masi L. D. E. Palma M. D. E. Lorito M. (2011). The beneficial effect of *Trichoderma* spp . on tomato is modulated by the plant genotype. *Mol. Plant Pathol.* 12 341–354. 10.1111/J.1364-3703.2010.00674.X 21453429 PMC6640367

[B74] van Lenteren J. C. Bolckmans K. Köhl J. Ravensberg W. J. Urbaneja A. (2017). Biological control using invertebrates and microorganisms: plenty of new opportunities. *BioControl* 63 39–59. 10.1007/s10526-017-9801-4

[B75] Vierheilig H. Schweiger P. Brundrett M. (2005). An overview of methods for the detection and observation of arbuscular mycorrhizal fungi in roots. *Physiol. Plant.* 125 393–404. 10.1111/j.1399-3054.2005.00564.x

[B76] Vitti A. Pellegrini E. Nali C. Lovelli S. Sofo A. Valerio M. (2016). Trichoderma harzianum T-22 induces systemic resistance in tomato infected by *Cucumber mosaic virus*. *Front. Plant Sci.* 7:1–11. 10.3389/fpls.2016.01520 27777581 PMC5056173

[B77] Whipps J. M. (2001). Microbial interactions and biocontrol in the rhizosphere. *J. Exp. Bot.* 52 487–511. 10.1093/jexbot/52.suppl_1.487 11326055

[B78] Wickham H. (2009). *ggplot2: Elegant graphics for data analysis*, 1st Edn. New York, NY: Springer-Verlag, 10.1007/978-0-387-98141-3

[B79] Wilson P. S. Ketola E. O. Ahvenniemi P. M. Lehtonen M. J. Valkonen J. P. T. (2008). Dynamics of soilborne *Rhizoctonia solani* in the presence of *Trichoderma harzianum*: effects on stem canker, black scurf and progeny tubers of potato. *Plant Pathol.* 57 152–161. 10.1111/j.1365-3059.2007.01706.x

[B80] Woo S. L. Ruocco M. Vinale F. Nigro M. Marra R. Lombardi N. (2014). *Trichoderma*-based products and their widespread use in agriculture. *Open Mycol. J.* 8 71–126. 10.2174/1874437001408010071

[B81] Zuur A. F. Ieno E. N. (2016). A protocol for conducting and presenting results of regression-type analyses. *Methods Ecol. Evolut.* 7 636–645. 10.1111/2041-210X.12577

