# Supplementary material for: Microbial Consortia for Effective Biocontrol of Root and Foliar Diseases in Tomato
Source: Front Plant Sci. 2021 Nov 5;12:756368. doi: 10.3389/fpls.2021.756368 (PMC8602810; doi:10.3389/fpls.2021.756368)
Supplement: Supplementary file 1 [file Data_Sheet_1.docx]

***Supplementary Material***

1. **Supplementary figures**


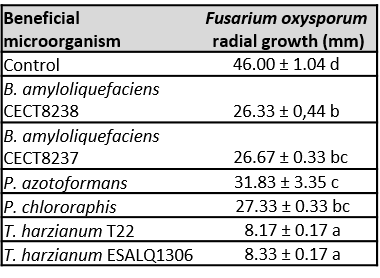


**A**

**B**

**Supplementary Figure 1.** **(A)** Soil samples from Control and SynCom2 treatments plated on TSA medium amended with natamycin for bacteria determination, and PDA medium amended with igepal and tetracycline for fungi determination. Pictures illustrate the appearance of *Pseudomonas* spp, *Bacillus* spp. and *Trichoderma* spp. colonies in SynCom2 treatment, and the absence of indigenous species from these genera in the control treatment. **(B)** Colonies of *P. azotoformans*, *B. amyloliquefaciens* CECT8238 and *T. harzianum* T22 after 72h of growth on PDA. Picture illustrates morphological differences between colonies.

**A**

| **Beneficial microorganism** | ***Fusarium oxysporum***  **radial growth (mm)** |
| --- | --- |
| Control | 46.00 ± 1.04 d |
| *B. amyloliquefaciens* CECT8238 | 26.33 ± 0,44 b |
| *B. amyloliquefaciens* CECT8237 | 26.67 ± 0.33 bc |
| *P. azotoformans* | 31.83 ± 3.35 c |
| *P. chlororaphis* | 27.33 ± 0.33 bc |
| *T. harzianum* T22 | 8.17 ± 0.17 a |
| *T. harzianum* ESALQ1306 | 8.33 ± 0.17 a |

**B**

| **Beneficial microorganism** | ***Botrytis cinerea***  **radial growth (mm)** |
| --- | --- |
| Control | 63.17 ± 0.44 g |
| *B. amyloliquefaciens* CECT8238 | 22.33 ± 0,44 d |
| *B. amyloliquefaciens* CECT8237 | 24.67 ± 0.44 e |
| *P. azotoformans* | 33.00 ± 0.76 f |
| *P. chlororaphis* | 15.33 ± 0.33 c |
| *T. harzianum* T22 | 7.33 ± 0.67 a |
| *T. harzianum* ESALQ1306 | 11.5 ± 0,29 b |

**Supplementary Figure 2.** In vitro confrontation assay of the selected microorganisms against the soil pathogenic fungus *Fusarium oxysporum* **(A)** and the leaf pathogenic fungus *Botrytis cinerea* **(B)**. For all plates, BCA on the left, pathogen on the right. Values are means of radial growth (mm) ± SE. Treatments not sharing a letter in common are significantly different based on general linear model and Tukey HSD test (p<0.05, n=3).

**Supplementary Figure 3.** Representative pictures of *B. cinerea* lesions in disease control and most effective BCA single strain (*P. chlororaphis*), and SynCom2B treatments, after foliar application.


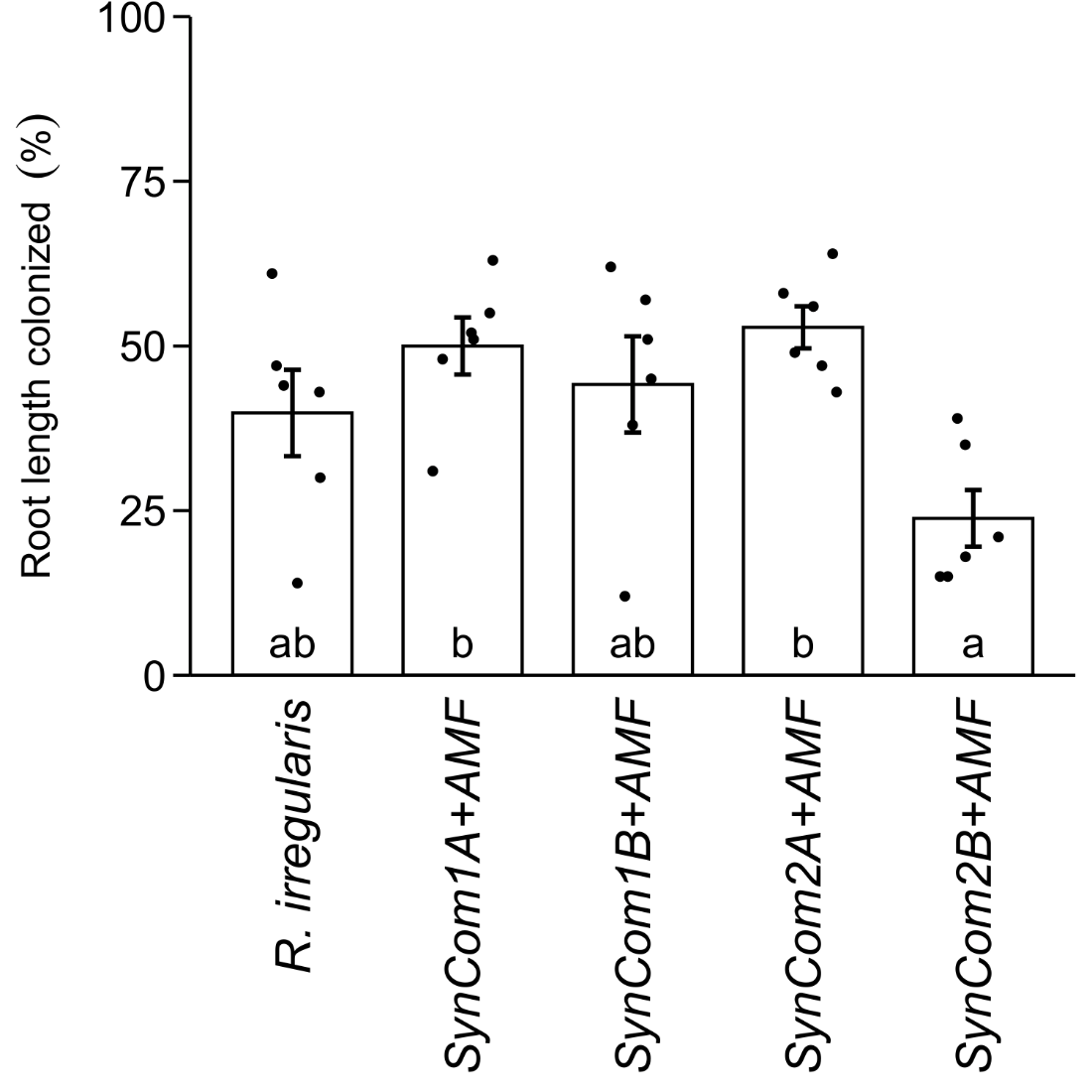


**Supplementary Figure 4.** Mycorrhizal colonization in plant roots from the *Botrytis* ISR bioassay represented as percentage of root length colonized by *R. irregularis.* Bars represent means ± SE. Treatments not sharing a letter in common are significantly different based on general linear model and Tukey HSD test (p<0.05; n=6).
